# Adherence to European Society of Cardiology guidelines at discharge after Acute Coronary Syndrome: a two-center data from Georgia

**DOI:** 10.1186/s43044-026-00721-y

**Published:** 2026-02-16

**Authors:** David Gogoberidze

**Affiliations:** 1 Tbilisi State University, Tbilisi, Georgia; 2 Tbilisi Heart Center, Tbilisi, Georgia

**Keywords:** Acute Coronary Syndrome, Secondary prevention, Adherence to guidelines, Discharge prescriptions

## Abstract

**Background:**

The aim of the study was to investigate the adherence to ESC guidelines in patients discharged after ACS in Georgia.

**Patients and methods:**

A prospective observational study was performed in 2 cardiac hospitals in Tbilisi from March 2021 till June 2022. A total of 428 consecutive patients with confirmed diagnosis of Acute Coronary Syndrome at discharge were evaluated. The primary outcome was defined as adherence to Guideline Directed Medical Therapy (GDMT) at discharge post-Acute Coronary Syndrome.

**Results:**

Complete guideline adherence for the combination of 5 medication classes was 74.5%, and additionally in 15.2% contraindications or intolerance were well documented, so the complete guideline adherence was very high − 89.7%. The guideline adherence for individual drug class differed from 98.9% for ACE inhibitors/ARB and acetylsalicylic acid to 92.9% for P2Y12 receptor inhibitor.

**Conclusion:**

Adherence to GDMT according to the data received from 2 hospitals is promising, but efforts to further improve guideline adherence must be targeted toward patient groups who receive the worse treatment at discharge, e.g. NSTEMI patients and only medical treatment sub-group.

## Background

According to the Global Burden of Disease (GBD) Study 2019 data, prevalent cases of total cardiovascular disease (CVD) nearly doubled from 1990 to 2019, and the number of CVD deaths increased from 12 million in 1990 to 18.1 million in 2019 [1]. In high-income countries, there has been a significant reduction in mortality from CVD over the last decades, but even there, CVD still remains a leading cause of death [2]. Georgia, a middle-income country with a population of up to 3.7 million, demonstrates a mean life expectancy of 71.4 years (75.4 for women and 67.5 for men) and 37% CVD mortality (the leader in mortality structure) according to 2021 data from the NCDC (National Center for Disease Control and Public Health) of Georgia [3]. Acute Coronary Syndrome (ACS) is one of the cornerstones of CVD and despite a marked improvement in in-hospital mortality of patients with ACS long-term outcome remains poor and the risk of recurrence of cardiovascular complications is 12–15% at 12 months [4]. Secondary prevention of CVD is strongly recommended by international guidelines. ESC (European Society of Cardiology) guidelines for the treatment of ACS (ST-segment Elevation Myocardial Infarction - STEMI, NSTEMI -non-ST-segment Elevation Myocardial Infarction, and UA -unstable angina) and CVD prevention allowed access to different evidence-based strategy[ 5,6,7], but there is a lack of data in Georgia that demonstrates the application of these guidelines to daily clinical practice and further follow up (FU) after discharge in patients with ACS. Regular use and optimal dosing with secondary prevention medications improve quality of life and survival reducing cardiovascular events and mortality by up to 80% [8]. The literature overview indicates that secondary prevention medications are inconsistently prescribed, commonly at sub-optimal doses, and poorly adhered to by patients. Statins can be initiated at optimal doses, while ACE/ARBs and beta-blockers need to be titrated to the optimal dose. Although optimizing doses before discharge is recommended, some patients may have contraindications or be unable to tolerate dose titration due to common factors such as hypotension, bradycardia, comorbidity, or short hospitalization duration, making such kind of optimization unrealistic. However, by ensuring patients are prescribed optimal secondary prevention medications at discharge, physicians can increase the likelihood of adherence to these medicines post-discharge and significantly improve long-term outcomes [9]. Studies evaluating current practices in Georgia against ESC guidelines (endorsed by the Georgian Society of Cardiology) are not available and this study aimed to investigate the current discharge treatment options in patients with ACS, focusing on ESC guidelines recommended 5 classes of medications: a combination of aspirin, P2Y_12_ receptor inhibitor, statin, beta-blocker and ACE-inhibitor (angiotensin-converting enzyme inhibitor) or ARB (angiotensin receptor blocker).

## Materials and methods

### Design

A prospective observational study was conducted in 2 cardiac hospitals in Tbilisi, Georgia from March 2021 to June 2022. Each participant’s information was collected from the hospital admission to discharge with the confirmed diagnosis of ACS.

### Population

Consecutively all adult patients discharged with the confirmed diagnosis of ACS (STEMI or NSTEMI) and UA were enrolled in the study. The main exclusion criteria were defined as: in-hospital mortality, transfer to another care facility, documented contraindications to guideline-recommended therapies pre-defined, cognitive impairment, incomplete or missing discharge medication data, non-ACS discharge diagnosis, participation in the other interventional clinical trial.

### Data collection

Information was collected from the hospital medical records and included: age, gender, previous CAD diagnosis (MI or coronary revascularization in medical history), presence of comorbidity, treatment strategies during hospitalization (coronary intervention or medical therapy), and prescriptions at discharge (including the dosing and frequency). The adherence was assessed using the discharge summary form, which includes all prescribed medications with its individual dosing instructions. This data enabled the definition of not only adherence at discharge for secondary prevention medication classes but also to calculate the compliance with target doses at discharge.

### Outcomes

The primary outcome of the study was to investigate the adherence to prescribing GDMT based on ESC guidelines for STEMI, NSTEMI and CV Prevention. Adherence to guidelines was defined as prescribing all 5 classes of medications: combination of aspirin, P2Y_12_ receptor inhibitor, statin, beta-blocker and ACE-inhibitor (angiotensin converting enzyme inhibitor) or ARB (angiotensin receptor blocker) unless intolerant or contraindicated.

The secondary outcome of the study was to assess the differences in discharge prescription between the ACS sub-groups (STEMI vs. NSTEMI and UA) and in-hospital treatment strategies (coronary intervention vs. medical treatment).

### Statistical analysis

Characteristics of the study population and guideline adherence were studied by means of descriptive statistics. Frequency and percentage analysis described the categorical variables, such as gender, comorbidities and types of ACS. Mean and standard deviation were used to describe the continuous variables (e.g., age). The variables were tested for normality using the Shapiro–Wilk test; no evidence of non-normal distribution was found.

The primary analysis was performed using adherence defined as prescribing all five classes of medications to all subjects unless contraindicated.

A chi-square test of independence was performed to examine the relationship between discharge diagnosis (predictor variable) (STEMI, NSTEMI, and UA) and adherence to guidelines.

A significant association was determined as *p* < 0.05.

The same chi-square test was performed to identify the significance of in-hospital treatment strategies as predictor variables and adherence to guidelines (PCI, CABG, or only medical treatment) with *p* < 0.05.

Statistical analysis was performed using SPSS (IBM SPSS statistics) version 29.0 (IBM Corp, Armonk, NY, USA).

## Results

In total, 500 patients were screened. Of these, only 428 were enrolled in the study. 38 refused to sign the informed consent form, 16 patients (3.7%) died during hospitalization. Furthermore, in 34 patients concomitant COVID-19 infection was diagnosed (the period of enrollment in the study was characterized by the massive spread of this infection in Georgia) and in 12 cases the diagnosis of ACS was not confirmed during the hospitalization. Patients’ clinical characteristics are described in Table 1.

**Table 1:** Patient’s clinical characteristics at discharge

Variable	*n* (%)
Age in years mean and standard deviation	61.1 + 7.3
Female	127 (29.7)
Discharge diagnosis	
STEMI	128 (29.9)
NSTEMI	163 (38.0)
UA	137 (32.0)
Type of treatment	
Medication	118 (27.6)
PCI	291 (68.0)
CABG	19 (4.4)
Comorbidity	
Hypertension	408 (95.3)
Diabetes mellitus	136 (31.8)
Prior Chronic Heart failure	129 (30.1)
CKD	64 (15.0)
PVD	48 (11.2)
COPD	12 (2.8)
Cardiac medical history	
Prior MI	144 (33.6)
Prior PCI	72 (16.8)
Prior CABG	24 (5.6)
Prior Angina pectoris	47 (11.0)

Complete guideline adherence for the combination of 5 classes of medications was 74.5%, and in addition, in 15.2% contraindications or intolerance was well documented, so the complete guideline adherence was very high − 89.7%. The guideline adherence for individual drug classes differed from 98.9% for ACE inhibitors/ARB and acetylsalicylic acid to 92.9% for P2Y_12_ receptor inhibitors. Table 2 represents the prescription rates for 5 classes of medications and guideline adherence.

**Table 2:** Prescription rates for 5 classes of medications and guidelines adherence

Drug	Prescription *n* (%)	Guideline adherence* *n* (%)
Acetylsalicylic acid	356 (83.1)	420 (98.3)
P2Y_12_ receptor inhibitor	342 (79.9)	398 (89.9)
Beta-blocker	344 (80.3)	408 (95.5)
ACE inhibitor or ARB	389 (90.8)	412 (96.3)
Statin	325 (76.1)	414 (96.9)
All 5 medications	319 (74.5)	383 (89.7)

*Guideline adherence includes the prescription of medication or documented contraindication

The compliance with guideline recommended target doses also was assessed in total study population and the results are shown in Table 3.

**Table 3:** Compliance with target doses at discharge

Drug	Prescription *n* (%)	At target/optimal dose *n* (%)*
Acetylsalicylic acid	356 (83.1)	356 (100)
P2Y_12_ receptor inhibitor	342 (79.9)	342 (100)
Beta-blocker	344 (80.3)	215 (63)
ACE inhibitor or ARB	389 (90.8)	148 (38)
Statin	325 (76.1)	284 (87)
All 5 medications	319 (74.5)	148 (46.1)

*Optimal dose is defined as ≥ 75% of guideline –recommended target dose

Adherence was also calculated taking in account the discharge diagnosis after ACS and the treatment strategy used during the hospitalization. Data are presented in Table 4.

**Table 4:** Patients’ diagnosis and treatment options’ association with guideline adherence

Variable	*n* (%)	Guideline adherence* *n* = 383 (%)	Guideline non-adherence *n* = 45 (%)	*P* value
Discharge diagnosis				
STEMI	128 (100)	123 (96.1)	5 (3.9)	p $\:\approx\:0.014$
NSTEMI	163 (100)	141 (86.5)	22 (13.5)	
UA	137 (100)	119 (86.9)	18 (13.1)	
Type of treatment				
Medication	118 (100)	86 (72.9)	32 (27.1)	*p* < 0.001
PCI	291 (100)	281(95.2)	10 (4.8)	
CABG	19	16 (84.2)	3 (15.8)	

Adherence rates differs across the 3 diagnoses groups, with STEMI with maximal adherence and UA less adherence. There is statistically significant association between the discharge diagnosis and treatment adherence, exact p value is 0.014.

Medication adherence differed significantly across treatment groups. Adherence was observed in 72.9% of patients receiving medication only, 96.6% undergoing PCI, and 84.2% undergoing CABG. The association between treatment strategy and adherence status was statistically significant (*p* < 0.001).

Sub-group analysis of patients and treatment strategy used for ACS management revealed a higher comorbidity burden in patients receiving only medication treatment. In this sub-group, the comorbidities were present with hypertension (97%), diabetes (48%), COPD (30%) and chronic kidney disease (12%) against the total study population already mentioned above. This sub-group was older, had higher prevalence of CAD and chronic heart failure in medical history.

Table 5 represents the treatment strategy used for ACS management during the hospitalization, showing the highest rate of intervention for STEMI (96.8%), followed by 78,5% for NSTEMI and 42.4% for UA.

**Table 5:** The treatment option split by diagnosis

Treatment Diagnosis	PCI *n* (%)	CABG *n* (%)	OMT *n* (%)
STEMI	118 (92)	6 (4.8)	4 (3.2)
NSTEMI	119 (73)	9 (5.5)	35 (21.5)
UA	54 (39.5)	4 (2.9)	79 (57.6)

## Discussion

This study is the first in Georgia performed to investigate adherence to prescribing GDMT to patients with ACS at hospital discharge. This study confirms that the 5-drug regimen was prescribed in both centers with sufficient frequency. Most of the cases when the GDMT was not adequately used were explained and intolerance or contraindications were documented. Most findings were associated with a specific drug class and mainly include well-known class effects like hypotension, the presence of concomitant diseases like COPD or CKD, historical data of major bleeding, or proven intolerance to high statin therapy.

Various already conducted studies gave us different information about the level of complete guideline adherence at discharge after ACS, this indicator varies from country to country and numbers are sometimes paradoxical [10, 11].

The results of this study confirmed that the adherence also varies from class to class of the drugs prescribed: with aspirin prescribed at the highest frequency and dual antithrombotic therapy (DABT) at the lowest. This finding is in line with already published studies [12].

It is conceivable that the high rate of adherence in this study can be explained by the specific clinical characteristics of the patients included:

1. Approximately 50% had already verified diagnosis of CAD and half of them were already revascularized.
2. Most of the patients had concomitant diseases in medical history per se requiring long-term treatment with ACE/ARB, BB, or statins (408 cases of arterial hypertension, 136 cases of Diabetes mellitus type 2, and 129 cases of heart failure).

It is well known that if the drug for secondary prevention is not prescribed at discharge from the hospital, the chance of its further use is quite low, so the high adherence obtained in this study is encouraging.

The issue and unmet need are whether the dosage of the drugs used was adequate and in compliance with the guideline recommendations. The analysis of dosing compliance (Table 3) highlights a significant gap between prescription rates and dose optimization. While Acetylsalicylic Acid was prescribed at target doses (100%), optimal dosing was achieved in only 63% of patients on beta-blockers and 38% on ACE inhibitors/ARBS. Consequently, only 46.1% of patients received full combination of all five drugs at optimal doses. The same problem, achieving target doses at discharge, was underlined in several publications before [13].

Discussing the secondary endpoints of this study and analyzing the adherence rate between the sub-groups of patients (STEMI vs. NSTEMI), it was identified that most of the patients hospitalized with ACS had a discharge diagnosis of NSTEMI vs. STEMI. This finding also differs from country to country according to the data published by the colleagues from Turkey and some other countries [14,15] and this data can be explained by statistics presented by NCDC Georgia in 2021 confirming the same tendencies throughout Georgia. It was found that the rate of use of guideline-recommended therapy in the STEMI subgroup was significantly higher compared to NSTEMI, which is consistent with data presented in other works [16.

Both centers participating in this study are located in Tbilisi, the capital of Georgia, and are fully equipped with coronary intervention facilities. No significant difference was found between the centers in the routine management of patients with ACS. Accordingly, all patients who required invasive intervention were given timely and adequate treatment. When the correlation between the adherence to guidelines and in-hospital treatment strategy was analyzed the discharge prescriptions showed significantly high guidelines adherence rate in the PCI group vs. only medical treatment.^17^.

The limitations of this study are the small sample size and its two-center design, with both centers located in the capital. In order to better generalize the results, it is very important to conduct more studies included the hospitals located all over Georgia.

The follow-up assessments beyond discharge were performed to assess the adherence to the guideline - recommended treatment compliance on 1, 6 and 12 months after ACS and its correlation with the rate of MACE after ACS. However long-term adherence and outcomes were not analyzed in this specific report.

## Conclusion

Adherence to GDMT according to the data received from two hospitals are promising, but efforts to further improve guideline adherence must be targeted on patient groups who receive the worse treatment at discharge, e.g., NSTEMI patients and the medical treatment sub-group.

## Data Availability

The data used and analyzed during the current study are available from the corresponding author on reasonable request.

## Abbreviations

ACS: Acute Coronary Syndrome
ACE: Angiotensin-converting enzyme
ARB: Angiotensin receptor blocker
BB: Beta-blocker
CABG: Coronary artery bypass grafting
CAD: Coronary artery disease
CKD: Chronic kidney disease
COPD: Chronic obstructive pulmonary disease
CV: Cardiovascular
DAPT: Dual antiplatelet therapy
ESC: European Society of Cardiology
GDMT: Guideline-directed medical therapy
IRB: Internal Review Board
LEC: Local Ethics Committee
MACE: Major adverse cardiovascular events
MI: Myocardial infarction
NSTEMI: Non-ST segment elevation myocardial infarction
PCI: Percutaneous coronary intervention
PVD: Peripheral vascular disease
STEMI: ST segment elevation myocardial infarction
UA: Unstable angina
